# Migration-Prevention Strategy to Fabricate Single-Atom Fe Implanted N-Doped Porous Carbons for Efficient Oxygen Reduction

**DOI:** 10.34133/2019/1768595

**Published:** 2019-08-22

**Authors:** Dong-Li Meng, Chun-Hui Chen, Jun-Dong Yi, Qiao Wu, Jun Liang, Yuan-Biao Huang, Rong Cao

**Affiliations:** ^1^State Key Laboratory of Structural Chemistry, Fujian Institute of Research on the Structure of Matter, Chinese Academy of Sciences, Fuzhou 350002, China; ^2^University of the Chinese Academy of Sciences, Beijing 100049, China

## Abstract

It is highly desired but challenging to achieve highly active single-atom Fe sites from iron-based metal-organic frameworks (MOFs) for efficient oxygen reduction reaction (ORR) due to the easy aggregation of iron species and formation of the inactive Fe-based particles during pyrolysis. Herein, a facile migration-prevention strategy is developed involving the incorporation of polyaniline (PANI) into the pores of iron porphyrinic-based MOF PCN-224(Fe) and followed by pyrolysis to obtain the single-atom Fe implanted N-doped porous carbons material PANI@PCN-224(Fe)-900. The introduced PANI inside the pores of PCN-224(Fe) not only served as protective fences to prevent the aggregation of the iron species during thermal annealing, but also acted as nitrogen sources to increase the nitrogen content and form Fe-N_x_-C active sites. Compared with the pristine PCN-224(Fe) derived carbonization sample containing Fe-based particles, the carbonaceous material PANI@PCN-224(Fe)-900 without inactive Fe-based particles exhibited superb ORR electrocatalytic activity with a more positive half-wave potential, significantly improved stability in both alkaline media, and more challenging acidic condition. The migration-prevention strategy provides a new way to fabricate atomically dispersed metal active sites via pyrolysis approach for promoting catalysis.

## 1. Introduction

The increase of environmental pollution and global energy demands promotes the development of the environmentally friendly energy conversion devices to avoid the use of fossil fuels [1–6]; among these, polymer electrolyte membrane fuel cell (PEMFC) and metal-air batteries can be as promising candidates in transportation vehicles and other power applications. However, the sluggish cathodic oxygen reduction reaction (ORR) has greatly hampered their development in commercial applications [7–9]. Although the platinum-based electrodes have been proved to be the most effective catalysts for ORR in both alkaline and acidic electrolytes [10–12], the high cost, scarcity, poor durability; and methanol-crossover hinder its commercialization process [11]. Thus, tremendous efforts have been devoted to develop nonprecious metal catalysts to replace the Pt-based electrodes [13–24]. Among of them, single-atom Fe anchored on N-doped porous carbons to form Fe-N_x_-C material is regarded as highly efficient active sites for ORR [16, 25–36]. Generally, the Fe-N_x_-C electrocatalysts are obtained by pyrolysis of precursors containing iron and nitrogen species [31, 37, 38]. However, the aggregation of iron species usually occurs during the pyrolysis process, which leads to the formation of large amounts of inactive aggregates, such as iron and Fe-C nanoparticles [28].

In recent years, metal-organic frameworks (MOFs) constructed by coordinate bonds between organic linkers and metal ions have attracted extensive attention in gas adsorption and catalysis because of their large surface areas, tunable structures, and pore sizes [39–42]. Particularly, heteroatoms are uniformed distributed in the highly ordered frameworks make MOFs as self-sacrificed precursors/templates for the fabrication of the highly efficient single-atom metal implanted carbon-based electrocatalysts [43, 44]. The unique structures of MOFs could endow the obtained electrocatalysts with large specific surface areas and the uniform distribution of heteroatom dopants [45–49]. However, the adjacent metal ions with no protective layer between the pores of MOF are usually prone to aggregate to form inactive metal-based nanoparticles during thermal pyrolysis. Therefore, it is imperative to develop a general approach to prevent the aggregation of metal species and fabricate active metal sites at atomic level for highly efficient ORR.

**Figure 1:**
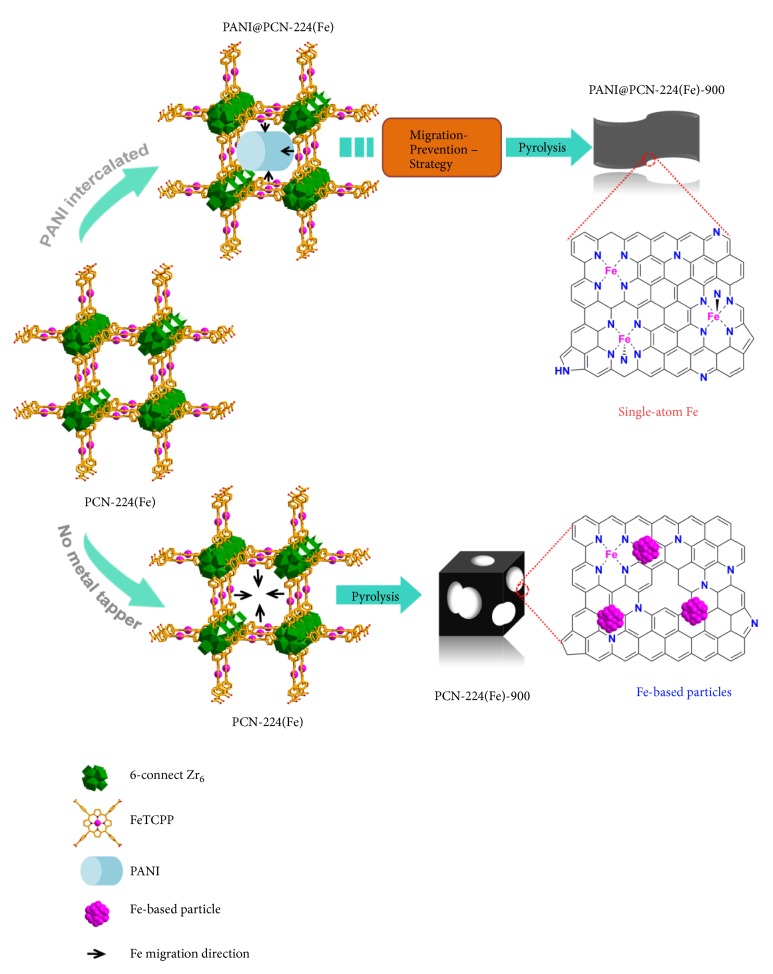
Schematic illustration of the fabrication of single-atom Fe catalyst PANI@PCN-224(Fe)-900 via migration-prevention strategy and the PCN-224(Fe) derived PCN-224(Fe)-900.

With the above in mind, a migration-prevention strategy was proposed to fabricate single-metal-atom catalyst via inhibition the aggregation of the metal species to form inactive metal-based particles (Figure 1). As a proof of concept, polyaniline (PANI) was in situ inserted in the pores of iron porphyrinic-based MOF PCN-224(Fe), [(Zr_6_O_4_(OH)_10_(H_2_O)_6_)(Fe-TCPP)_1.5_]_n_ (Fe-TCPP = [5,10,15,20-Tetrakis(4-carboxyphenyl)porphyrinato]-Fe(III) Chloride) [50]. During pyrolysis process, the PANI in the pores of PCN-224(Fe) as protective fence can prevent the aggregation of the adjacent iron species between the pores. Interestingly, PANI containing nitrogen-rich element could not only promote the formation of the N-doped carbon sites, but also capture the iron species and stabilize the atomically dispersed Fe-N_x_ active sites. Thus, owning to the “partition role” of PANI, the obtained carbonaceous material PANI@PCN-224(Fe)-900 has abundant single-atom Fe active sites, large surface area, high conductivity, and nitrogen content. It exhibited highly efficient ORR activity and ultrahigh stability, far surpassing the pristine PCN-224(Fe) derived carbon materials PCN-224(Fe)-900 in both alkaline and acidic media. Moreover, the extremely good ORR performance of PANI@PCN-224(Fe)-900 with a half-wave potential (*E*_1/2_ = 0.893 V) also outdistanced the commercial Pt/C catalysts (*E*_1/2_ = 0.856 V) under alkaline conditions. More importantly, it exhibited comparable half-wave potential (*E*_1/2_ = 0.756 V) to that of the state-of-the-art Pt/C (0.783 V) in more challenging acidic media.

## 2. Results and Discussion

**Figure 2:**
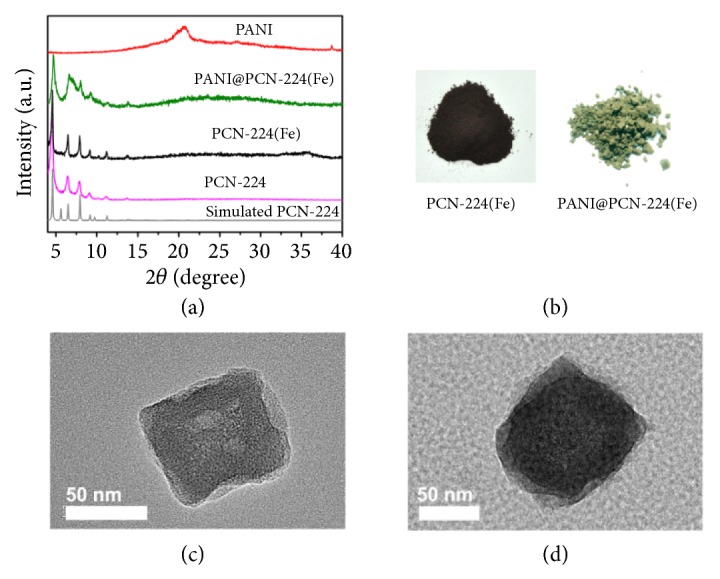
(a) PXRD patterns of PCN-224, PCN-224(Fe), PANI@PCN-224(Fe), and PANI. (b) Optical images of PCN-224(Fe) and PANI@PCN-224(Fe) powder. TEM images of (c) PCN-224(Fe) and (d) PANI@PCN-224(Fe).

As we know, PANI is usually synthesized by oxidizing aniline using ammonium peroxodisulfate as catalyst [51]. However, it is difficult to completely remove the catalysts residual from the system, which could in turn bring negative impact on the sensitive electrocatalytic system [52]. Therefore, PCN-224(Fe) was selected as host to encapsulate PANI, in which the iron(III) porphyrin moieties in the pore wall (Figures [Supplementary-material supplementary-material-1] and [Supplementary-material supplementary-material-1]) can catalyze the oxidative polymerization of aniline to form PANI with the aid of the environmentally friendly cocatalyst H_2_O_2_ [53]. The self-catalysis strategy is no need to use of any other catalyst that is difficult for removal in the MOF, thus avoiding the introduction of residues to affect the electrocatalysis. The iron ions were coordinated by the porphyrin units in PCN-224 by using postsynthesis method (PSM). The powder X-ray diffraction (PXRD) patterns of PCN-224(Fe) were not changed in comparision with those of PCN-224 (Figure 2(a)). After PSM treatment, the obtained PCN-224(Fe) still has high Brunauer–Emmett–Teller (BET) surface area of 898 m^2^g^−1^ ([Supplementary-material supplementary-material-1]) and large hydrophilic pore (1.8 nm) ([Supplementary-material supplementary-material-1]), which facilitates the adsorption of aniline. To investigate the migration-prevention effect, the different loading amounts of PANI in the pores of PCN-224(Fe) were controlled by the adsorption time and as high as 27 wt% PANI can be inserted into its channels after 12 h adsorption. The obtained composites are designated as* X*-PANI@PCN-224(Fe) (*X* = 15% and 27%), where* X* is the mass fraction of PANI. Notably, PANI@PCN-224(Fe) was referred to as 27%-PANI@PCN-224(Fe) in this article. For comparison, PANI was isolated from PANI@PCN-224(Fe) by digestion of PCN-224(Fe) with strong alkali solution.

After polymerization, the brown PCN-224(Fe) was become to olive-green (Figure 2(b)), indicating that PANI had been inserted in the pores of PCN-224(Fe). The successful insertion of PANI into PCN-224(Fe) was further proved by FT-IR spectra and UV-Vis diffuse reflectance spectra. As shown from the FT-IR spectra in [Supplementary-material supplementary-material-1], the bands at 1586 and 1489 cm^−1^ are attributed to quinone and benzine ring deformation of PANI, respectively [53], while the bands at 1300 and 1240 cm^−1^ are due to C-N and C=N stretching of the secondary aromatic amine. The peaks of quinonoid and benzenoid units of PANI in PANI@PCN-224(Fe) were observed at 1150 cm^−1^ and 820 cm^−1^, respectively. The UV-Vis spectra of the isolated PANI, PANI@PCN-224(Fe), and PCN-224(Fe) are displayed in [Supplementary-material supplementary-material-1]. The peaks at 322 and 931 nm in PANI@PCN-224(Fe) were contributed by *π* → *π∗* and *π*→ polaron band transitions of PANI [54]. Moreover, the peaks at 424, 501, and 618 nm are corresponding to Soret band and Q band of iron porphyrin motifs in PANI@PCN-224(Fe), suggesting that the iron porphyrin based framework was stable during the self-catalysis synthesis of PANI. The PXRD patterns of PANI@PCN-224(Fe) (Figure 2) are coincident with those of PCN-224(Fe), further indicating that the structure of PCN-224(Fe) was still maintained during the polymerization process. Moreover, the sharp decrease of BET surface area (271 m^2^g^−1^) and pore size (with a diameter of ~ 0.66 and 1 nm) of PANI@PCN-224(Fe) after the loading of PANI (Figures [Supplementary-material supplementary-material-1] and [Supplementary-material supplementary-material-1]) suggested that most of pores have been filled by PANI. The TEM images show that the cubic PANI@PCN-224(Fe) appeared a much darker contrast than PCN-224(Fe) (Figures 2(c) and 2(d)), further indicating that the pores have been occupied by PANI.

**Figure 3:**
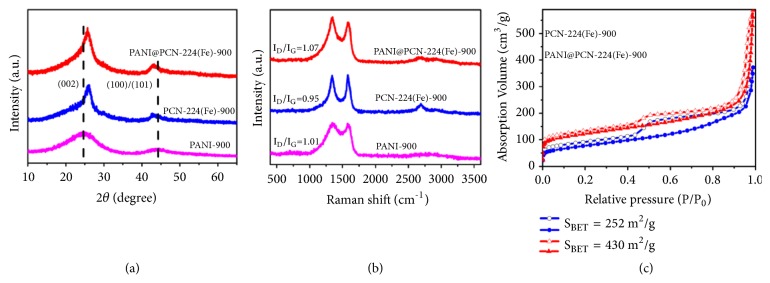
(a) PXRD, (b) Raman spectra of PANI@PCN-224(Fe)-900, PCN-224(Fe)-900, and PANI-900. (c) N_2_ adsorption-desorption isotherms of PANI@PCN-224(Fe)-900 and PCN-224(Fe)-900, while PANI-900 has no N_2_ adsorption.

After thermo-treatment at 900°C, no identifiable peak of Fe-based particles was observed in the PXRD ([Supplementary-material supplementary-material-1]) of PANI@PCN-224(Fe)-900 before acid etching. By contrast, the peaks of Fe and Fe_3_C particles were detected in PCN-224(Fe) derived materials PCN-224(Fe)-900. The results suggested that the inserted PANI in PANI@PCN-224(Fe)-900 indeed plays a protective fence role in preventing the aggregation of Fe elements during the pyrolyzing process. After removal of ZrO_2_ by HF etching, two apparent broad peaks centered at about 26° and 43° are observed in both of the PXRD patterns of PCN-224(Fe)-900 and PANI@PCN-224(Fe)-900, indicating that high degree of graphitic carbon with (002) and (100)/(101) diffractions was obtained (Figure 3(a)). The Raman spectra show (Figure 3(b)) two characteristic carbon resonances assigned to the G-band (1600 cm^−1^) and D-band (1350 cm^−1^), which correspond to graphitic sp^2^-hybridized carbon and sp^3^ carbon, respectively [55]. The relatively higher intensity ratio (*I*_D_/*I*_G_) of PANI@PCN-224(Fe)-900 was compared with PCN-224(Fe)-900, suggesting abundant carbon defective active sites were generated due to the introduction of N-riched PANI. Electrochemical impedance (EIS) measurement showed that the minimum semicircle of the Nyquist plots for PANI@PCN-224(Fe)-900 is observed, suggesting the best conductivity among the carbonaceous materials ([Supplementary-material supplementary-material-1]). This is because the fact that the inserted PANI backbone throughout PCN-224(Fe) channels could prevent the pores collapse and the PANI-derived carbon can behave as interconnection for electrons transportation between internal surfaces [56]. Thus, as shown in Figure 3(c), PANI@PCN-224(Fe)-900 shows larger N_2_ adsorption uptake and 1.7 times higher BET surface area (430 m^2^g^−1^) in comparison with PCN-224(Fe)-900 (252 m^2^g^−1^). In addition, both PCN-224(Fe)-900 and PANI@PCN-224(Fe)-900 have obvious desorption hysteresis loops, indicating mesopores were produced ([Supplementary-material supplementary-material-1]), which were favorable for the diffusion of the electrolyte and accessible to the active sites.

**Figure 4:**
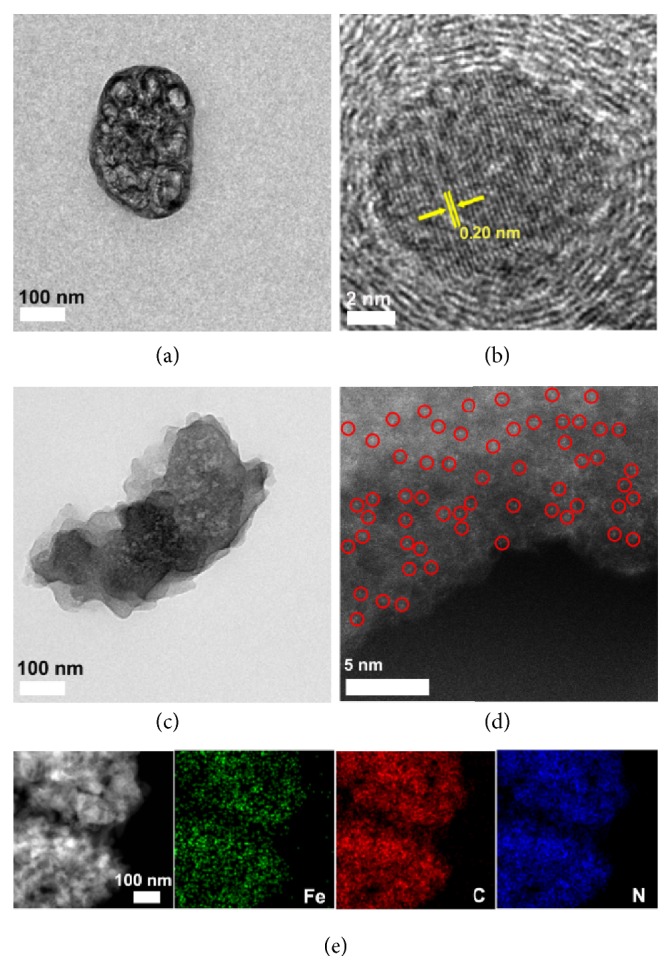
(a) TEM images and (b) HRTEM images of PCN-224(Fe)-900. (c) TEM images and (d) aberration-corrected HAADF-STEM image of PANI@PCN-224(Fe)-900. (e) HAADF-STEM image and corresponding Fe, C, and N elemental mappings of PANI@PCN-224(Fe)-900.

As shown in Figure 4(a), the TEM images reveal that, after pyrolysis, the cubic PCN-224(Fe) (Figure 2(c) and [Supplementary-material supplementary-material-1]) becomes elliptical PCN-224(Fe)-900 with inconsecutive mesoporous structures, indicating the skeleton collapse happened upon pyrolysis. In contrast, PANI@PCN-224(Fe)-900 exhibits denser structure containing smaller pores due to the inserted PANI-derivated carbons (Figure 4(c)). Furthermore, Fe_3_C/Fe nanoparticles (NPs) with a lattice distance of 0.20 nm can be observed in the high-resolution TEM image (HRTEM, Figure 4(b)) of PCN-224(Fe)-900, while no visible Fe-based NP was detected in PANI@PCN-224(Fe)-900 (Figure 4(c), [Supplementary-material supplementary-material-1]). The aberration-corrected HAADF-STEM revealed single Fe atoms distributed in carbonaceous matrix, as the atomic dispersed bright spots were detected (Figure 4(d)). Further inspection by elemental mapping (Figure 4(e)), Fe and N elements are homogenously distributed throughout the whole carbonaceous matrix of PANI@PCN-224(Fe)-900. The results were in consistent with the PXRD ([Supplementary-material supplementary-material-1] and Figure 3(a)) and Raman spectra (Figure 3(b)). This is because the fact that the inserted PANI could form nonporous carbon with densely stacked 2D layer structure ([Supplementary-material supplementary-material-1]), which could behave as a promising protective fence to prevent the migration of the iron species to form Fe NPs during thermal annealing. The HRTEM image shows ([Supplementary-material supplementary-material-1]) abundant graphitic carbon crystalline structure is presented in PANI@PCN-224(Fe)-900, as the distinct lattice fringes with an interplanar space of 0.34 nm corresponding to the (002) plane of graphite can be observed. Such highly graphitic structure could facilitate the electron transport and thus will improve electrocatalytic activity.

**Figure 5:**
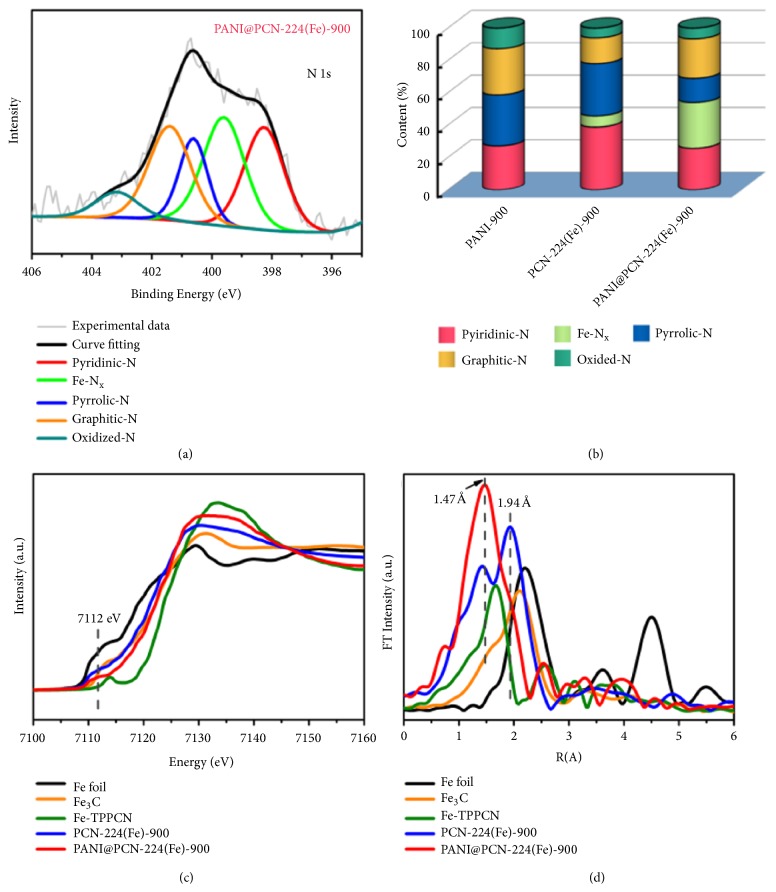
(a) High-resolution N 1s spectra of PANI@PCN-224(Fe)-900. (b) Nitrogen configurations of PANI-900, PCN-224(Fe)-900 and PANI@PCN-224(Fe)-900. (c) Fe K-edge XANES spectra and (d) Fourier transform EXAFS of PANI@PCN-224(Fe)-900, PCN-224(Fe)-900, Fe foil, Fe-TPPCN, and Fe_3_C.

X-ray photoelectron spectroscopy (XPS) measurements also demonstrated that both of PCN-224(Fe)-900 and PANI@PCN-224(Fe)-900 samples contain Fe, N, and C elements ([Supplementary-material supplementary-material-1]). The high-resolution N 1s spectra can be deconvoluted into pyridinic N (398.2 eV), Fe-Nx (399.4 ± 0.2 eV), pyrrolic N (400.5 ± 0.1 eV), graphitic N (401.4 ± 0.2 eV), and oxidized N (403.2 ± 0.2 eV) (Figure 5(a) and Figures [Supplementary-material supplementary-material-1] and [Supplementary-material supplementary-material-1]) [57]. Notably, the relative ratio of Fe-Nx with other nitrogen species in PANI@PCN-224(Fe)-900 is much higher than that of PCN-224(Fe)-900 (Figure 5(b)). For iron species, two peaks at 710 and 721.9 eV in the high-resolution Fe 2p spectrum of PANI@PCN-224(Fe)-900 suggested that Fe^2+^ species was dominated in this sample, which may be coordinated with nitrogen that inherited from Fe-TCPP ([Supplementary-material supplementary-material-1]) [27, 28, 58]. Furthermore, according to the elemental analysis (EA, [Supplementary-material supplementary-material-1]) and inductively coupled plasma atomic emission spectroscopy (ICP-AES, [Supplementary-material supplementary-material-1]) results, both of the N and Fe element contents in* X*-PANI@PCN-224(Fe)-900 increase along with the increasing of the inserted PANI content. It is also worth mentioning that PANI@PCN-224(Fe)-900 has much higher amounts of N and Fe element than those of PCN-224(Fe)-900 (Tables [Supplementary-material supplementary-material-1] and [Supplementary-material supplementary-material-1] and [Supplementary-material supplementary-material-1]). The results benefit from the fact that the PANI in PANI@PCN-224(Fe)-900 could provide effective protective layers and prevent the Fe species migrating and aggregating to form particles during pyrolysis. Moreover, the escaped iron element from Fe-TCPP units during annealing could be captured by the inserted PANI-derived N-doped carbon to stabilize atomic Fe-N_x_ species [25, 38, 59–61]. Thus, PANI@PCN-224(Fe)-900 has a higher Fe amount of 1.89 wt%, while only 0.27 wt% Fe content comprising part of Fe_3_C/Fe was detected in PCN-224(Fe)-900 ([Supplementary-material supplementary-material-1] and Figure 4(b)). The very low Fe amount in PCN-224(Fe)-900 is due to the Fe ions which were lack of protection and easy formed aggregated Fe-based NPs, which were leached by acid etching. In addition, the inserted PANI could also act as nitrogen sources to increase the nitrogen content and nitrogen-containing carbon active-site density of PANI@PCN-224(Fe)-900 (Figures [Supplementary-material supplementary-material-1] and [Supplementary-material supplementary-material-1]), which could enhance synergistically for ORR in combination with Fe-N_x_ sites [38, 62, 63]. To further identify the electronic and structural status of iron species in PANI@PCN-224(Fe)-900 and PCN-224(Fe)-900, X-ray absorption near-edge structure (XANES) and X-ray absorption fine structure (EXAFS) were performed (Figures 5(c) and 5(d)). The absorption edge energy of Fe K-edge in PANI@PCN-224(Fe)-900 and PCN-224(Fe)-900 located between Fe foil and 5,10,15,20-tetrakis(4-cyanophenyl)porphyrinato]-Fe(III) chloride (Fe-TPPCN), implying the positively charged iron species in both of the two samples, which are consistent with the XPS results. Furthermore, a weak pre-edge peak at 7112 eV was observed in both of PANI@PCN-224(Fe)-900 and PCN-224(Fe)-900, which is recognized as a fingerprint of *D*_4h_ symmetry and suggested the existence of Fe-N_4_ square-planar structure [25, 33, 64]. The analysis of Fe K-edge of EXAFS further revealed that atomic Fe-N_x_ species was predominated in PANI@PCN-224(Fe)-900, while PCN-224(Fe)-900 contains the N-coordinated Fe and a large amount of Fe-based NPs including Fe_3_C and Fe. As shown in Figure 5(d), the main signal at 1.47 Å that assigned to the Fe-N scattering path further revealed that the Fe species in PANI@PCN-224(Fe)-900 was predominant Fe-N_x_ configuration [32, 65]. Notably, compared with the R value (1.47 Å) in PANI@PCN-224(Fe)-900, the corresponding value of Fe(Cl)-N_4_ peak in Fe-TPPCN molecule shifts to 1.59 Å, which is ascribed to that the axial chlorine atom perpendicular to the Fe-N_4_ plane elongates the Fe-N bond distance [25, 26, 31, 66–68]. Nevertheless, the partial R values overlap between PANI@PCN-224(Fe)-900 and Fe-TPPCN suggests the existence of Fe-N_4_ and Fe-N_5_ species, which is difficult to use suitable coordination model to fit. The emergence of Fe-N_5_, in which one axial nitrogen atom is perpendicular to the Fe-N_4_ plane, was attributed to the nitrogen of the inserted PANI-derived carbon. More importantly, no obvious signals ascribed to Fe-Fe (2.2 Å) can be detected, manifesting the atomic dispersion of iron in PANI@PCN-224(Fe)-900. By contrast, the EXAFS curves of PCN-224(Fe)-900 exhibited a strong peak at 1.94 Å, which is more like the Fe-Fe distance in Fe_3_C and metallic Fe [27, 30]. These results confirmed that Fe-based NPs were presented in PCN-224(Fe)-900, which is consistent with the high-resolution TEM observation (Figure 4(b)). Taken together, the highly porous PANI@PCN-224(Fe)-900 with graphitic structure contains abundant single-atom Fe-N_x_ sites, which is believed to show highly active in electrocatalysis for ORR.

**Figure 6:**
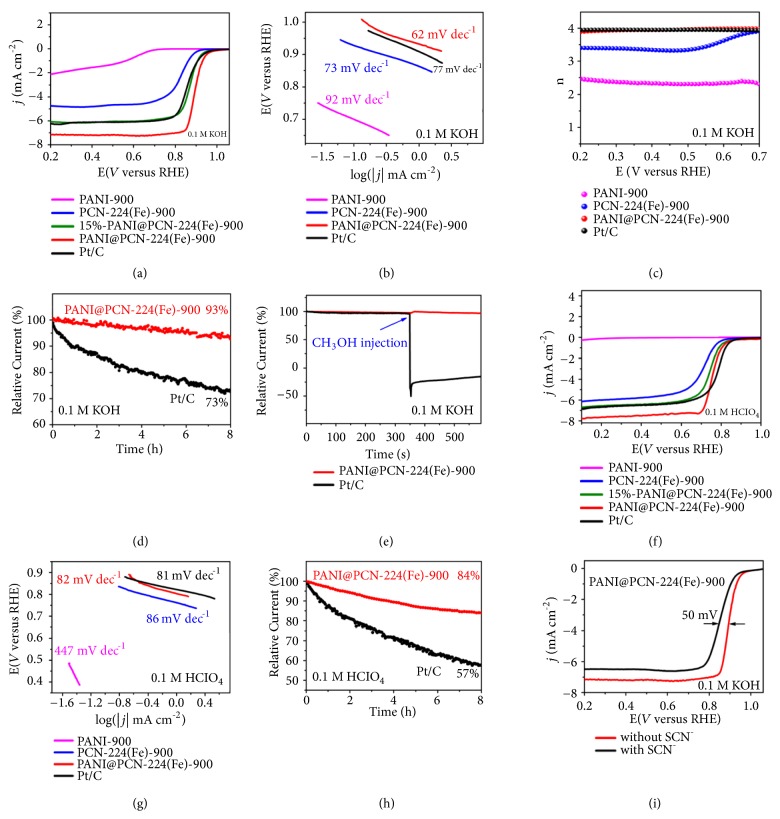
(a) LSVs of different samples at 1600 rpm and (b) the corresponding Tafel plots obtained from the RDE polarization curves in 0.1 M KOH. (c) Electron transfer number of different samples obtained from the RRDE curves in 0.1 M KOH. (d) Stability test of PANI@PCN-224(Fe)-900 in 0.1 M KOH. (e) Methanol-crossover effects test of PANI@PCN-224(Fe)-900 in 0.1 M KOH. (f) LSVs of different samples at 1600 rpm and (g) the corresponding Tafel plots obtained from the RDE polarization curves in 0.1 M HClO_4_. (h) Stability test of PANI@PCN-224(Fe)-900 in 0.1 M HClO_4_. (i) LSVs of PANI@PCN-224(Fe)-900 before and after the addition of 0.1 M SCN^−^ in 0.1 M KOH.

To assess the electrocatalytic performance of PANI@PCN-224(Fe)-900, ORR was firstly investigated on a rotating disk electrode (RDE) in O_2_-saturated 0.1 M KOH. As shown in Figure 6(a), the linear sweep voltammetry (LSV) curves reveal that PANI@PCN-224(Fe)-900 has a more positive half-wave potential (*E*_1/2_ = 0.893 V vs. RHE, all the potentials are referenced with RHE), 70 mV higher than PCN-224(Fe)-900 (*E*_1/2_ = 0.823 V), even 37 mV higher than the commercial 20 wt% Pt/C (*E*_1/2_ = 0.856 V, Alfa Aesar), indicating that the single Fe atoms contribute much to the ORR performance of PANI@PCN-224(Fe)-900. The influence of PANI content on ORR activity was also studied (Figure 6(a)). 15%-PANI@PCN-224(Fe)-900 displays comparable ORR performance (*E*_1/2_ = 0.870 V) with Pt/C, much better than PCN-224(Fe)-900, although it was less well-behaved in comparison with PANI@PCN-224(Fe)-900. It is due to the introduction of PANI, which acted as protective fence to increase to amount of Fe single-atom. A sufficient quantity of Fe single-atom active sites was generated; it is enough for PANI@PCN-224(Fe)-900 to surpass the state-of-the-art Pt/C. Thus, among the four samples, PANI@PCN-224(Fe)-900 displays the most positive onset potential *E*_onset_  (1.01 V), which far outdistanced those of 15%-PANI@PCN-224(Fe)-900 (0.977 V), PCN-224(Fe)-900 (0.94 V), Pt/C (0.973 V), and PANI-900 (0.75 V). Moreover, PANI@PCN-224(Fe)-900 shows the largest diffusion-limiting current density of 7.17 mA cm^−2^ at 0.2 V vs. RHE with 1600 rpm among the related catalysts, confirming its superb electrocatalytic activity. The excellent ORR activity of PANI@PCN-224(Fe)-900 was also verified by the smallest Tafel slope (62 mV dec^−1^, Figure 6(b)), in comparison with those of PCN-224(Fe)-900 (73 mV dec^−1^), Pt/C (77 mV dec^−1^), and PANI-900 (92 mV dec^−1^). The electron transfer number (n) of PANI@PCN-224(Fe)-900 calculated according to rotating ring-disk electrode (RRDE) is 3.95 (*E* = 0.2 V), which is similar to that of Pt/C (n = 3.95), suggesting an efficient four-electron ORR pathway (Figure 6(c)). The methanol-crossover effects and durability of PANI@PCN-224(Fe)-900 were also examined. After 8 h of continuous polarization (Figure 6(d)), only 7% current density loss was observed in PANI@PCN-224(Fe)-900, while 27% current decrease was observed for Pt/C, which exemplified the good durability of PANI@PCN-224(Fe)-900. Additionally, PANI@PCN-224(Fe)-900 also demonstrated a superior tolerance against crossover effect of methanol under alkaline condition, which was much better than the Pt/C (Figure 6(e) and [Supplementary-material supplementary-material-1]). It is worth noting that PANI@PCN-224(Fe)-900 possesses one of the best ORR performance among all the reported nonnoble metal catalysts under alkaline condition ([Supplementary-material supplementary-material-1]). Encouraged by the excellent ORR performance of PANI@PCN-224(Fe)-900 in alkaline media, we further explored its ORR activity in the more challenging acidic condition (0.1 M HClO_4_). Surprisingly, a more positive* E*_1/2_ of 0.756 V for PANI@PCN-224(Fe)-900 was achieved (Figure 6(f)), which is much more than PANI-900 (no active), 15%-PANI@PCN-224(Fe)-900 (0.74 V), and PCN-224(Fe)-900 (0.707 V), even comparable to that of benchmark Pt/C (0.783 V). Furthermore, PANI@PCN-224(Fe)-900 has the largest diffusion-limiting current density (7.78 mA cm^−2^, Figure 6(d)) at 0.1 V vs. RHE, which is higher than those of PCN-224(Fe)-900 (6.14 mA cm^−2^) and Pt/C (6.84 mA cm^−2^). Moreover, the small Tafel slope (82 mV dec^−1^, Figure 6(g)) of PANI@PCN-224(Fe)-900 is comparable to that of Pt/C (81 mV dec^−1^) and an ideal 4e^−^ transfer process (n = 3.93, [Supplementary-material supplementary-material-1]) based on RRDE further manifests the superior ORR activity of PANI@PCN-224(Fe)-900. Moreover, PANI@PCN-224(Fe)-900 exhibited long-term durability, in contrast with Pt/C, which showed poor stability in acid media (Figure 6(h)). The much better tolerance to methanol performance for PANI@PCN-224(Fe)-900 in comparison with Pt/C was also demonstrated the advantage of the single-atom Fe-N_x_ sites ([Supplementary-material supplementary-material-1]). Notably, the ORR activity of PANI@PCN-224(Fe)-900 in acidic media is also one of the best reported nonprecious metal catalysts ([Supplementary-material supplementary-material-1]).

The above results suggested that the atomically dispersed Fe-N_x_ is believed to be the highly active sites in PANI@PCN-224(Fe)-900 for ORR. The Fe-free catalyst PCN-224-900 showed very poor activity for the ORR, especially in acidic media ([Supplementary-material supplementary-material-1]), indicating that the activity of PANI@PCN-224(Fe)-900 catalyst mainly stems from Fe site. The SCN^−^ poisoning experiment was further carried out to confirm the active site, as SCN^−^ displays strong affinity to Fe ion. As shown in Figure 6(i), the diffusion-limiting current density of PANI@PCN-224(Fe)-900 for ORR in 0.1 M KOH decreased obviously from 7.17 mA cm^−2^ to 6.47 mA cm^−2^ (0.2 V vs. RHE), while the half-wave potential decreased significantly by 50 mV. Thus, the poisoning experiment clearly revealed that Fe-N_x_ was verified to be the active site in PANI@PCN-224(Fe)-900 for ORR.

## 3. Conclusion

In summary, we have developed a facile migration-prevention strategy to fabricate Fe single-atom anchored on N-doped porous carbons to boost oxygen reduction reaction. The nitrogen-riched polymer PANI was in situ synthesized and inserted in the channels of PCN-224(Fe) catalyzed by the Fe-TCPP active sites in the pore walls. The inserted PANI-derived N-doped carbon was acted as protective fences to prohibit the aggregation of iron species to form inactive Fe-based NPs during the pyrolysis process. Moreover, the nitrogen element in the PANI-derived N-doped carbon could be behaved as a metal trapper to capture the escaped iron from Fe-TCPP units and stabilize Fe-N_x_ species. Benefiting from the introduced PANI, the PANI@PCN-224(Fe)-900 has abundant single-atom Fe-N_x_ sites, high content of nitrogen, large surface area, and good conductivity. Thanks to the above-mentioned unique features, PANI@PCN-224(Fe)-900 turns out to be one of the most excellent ORR catalyst in both alkaline and acidic electrolyte, even surpassing commercial Pt/C. Our work opens a new avenue to prepare single-atom metal catalysts for boosting their electrocatalytic performances in practical energy storage and conversion applications.

## 4. Materials and Methods

### 4.1. Synthesis of Catalysts

*Synthesis of PCN-224(Fe)*. H_2_TCPP [69] and the nanoscale PCN-224 [70] were prepared according to the literatures. The nanoscale PCN-224(Fe) was prepared by a postsynthesis method based on the reported procedure [50]. Typically, the nanoscale PCN-224 (200 mg) and 500 mg FeCl_2_·4H_2_O were dispersed in DMF and heated at 120°C with stirring for 12 h. After that, the mixture was centrifuged and washed with fresh DMF, acetone twice, respectively. After removing acetone, the sample was dried in an oven at 70°C for 6 h. The whole process was tracked by PXRD.

*Synthesis of PANI@PCN-224(Fe)*. The nanoscale PCN-224(Fe) (300 mg) was degassed in a Schlenk vial for 12 h at 150°C. Then, aniline hydrochloride (200 mg) dissolved in H_2_O (1 mL) was introduced and sonicated for 15 min. The Schlenk vial was then kept in refrigerator (ca. 4°C) for different duration time (5 h and 12 h). After that, 8 mL diluted H_2_O_2_ (0.02 M) was added dropwise into the Schlenk vial and kept under stirring at 0°C for 24 h. Then the product was obtained by centrifugation and washing with acetone for 5 times. Finally, the composite denoted as* X*-PANI@PCN-224(Fe) was dried under vacuum overnight, where* X* is the mass fraction of PANI in PCN-224(Fe) which varied from 15% to 27% depending on adsorption time (5 h and 12 h, respectively).* X* was determined by weighing the extracted isolated PANI from* X*-PANI@PCN-224(Fe). For convenience, PANI@PCN-224(Fe) was referred as 27%-PANI@PCN-224(Fe) in this article.

*Preparation of Isolated PANI*. The PANI in* X*-PANI@PCN-224(Fe) was extracted out by successively immersing the* X*-PANI@PCN-224(Fe) composite powder in aqueous NaOH solution (2 M) for 12 h and obtained by rinsing with water and dried under vacuum at 120°C.

*Preparation of PCN-224-900, PCN-224(Fe)-900, PANI@PCN-224(Fe)-900, and PANI-900*. A typical procedure was as follows:* X*-PANI@PCN-224(Fe) (*X* = 15%, 27%) powder (200 mg) was placed in a ceramic boat and transferred to a tube furnace, then heated to 200°C with a heating rate of 5°C min^−1^ and remained at 200°C for 2 h, then heated to 900°C with a heating rate of 5°C min^−1^, and calcined for 2 h at 900°C under nitrogen. After that, to remove the formed ZrO_2_ in the carbonaceous material, the calcined sample was etched using 5% HF solution three times at room temperature for 12 h and washed thoroughly with water and ethanol, respectively. The obtained sample was dried in vacuum at 120°C for 12 h before use. The as-prepared samples were designated as* X*-PANI@PCN-224(Fe)-900 (*X* = 15%, 27%). For convenience, PANI@PCN-224(Fe)-900 was referred as 27%-PANI@ PCN-224(Fe)-900 in this article. The PCN-224-900, PCN-224(Fe)-900, and PANI-900 samples were prepared as the same procedure except using PCN-224, PCN-224(Fe), and the isolated PANI as precursors, respectively.

### 4.2. Oxygen Reduction Reaction (ORR) Measurements

All the electrochemical experiments were carried out on an IM6ex (Zahner, Germany) electrochemical station. Typically, 5 mg of the catalyst and 40 *μ*L Nafion (5% in lower ahatic alcohols and water) were dispersed in 1 mL ethanol under ultrasonication for 60 min. Then the as-prepared catalyst ink (5 *μ*L) was dropped onto the surface of a rotating disk electrode (RDE) with a glassy carbon (GC) disk of 5 mm in diameter, after dried in air, the operation was repeated 3 times; totally 20 *μ*L ink was dropped on GC disk. The commercial 20 wt% Pt/C (Alfa Aesar) electrode was prepared by dispersing 2.5 mg of the catalyst in 1 mL deionized water and totally 20 *μ*L ink was dropped on GC disk. All electrochemical measurements were performed in a conventional three-electrode setup with RDE of 5 mm in diameter coated with catalysts as the working electrode, an Ag/AgCl electrode as the reference electrode and a graphite rod as the counter-electrode in 0.1 M KOH or HClO_4_ electrolyte. Prior to the electrochemical measurements, the electrolyte solution was saturated with O_2_ by bubbling O_2_ for 30 min. In order to ensure the O_2_ saturation, the O_2_ flow was maintained during the measurements. The polarization curves were recorded after 20 cycles of cyclic voltammetry (CV) curves with a scan rate of 10 mV·s^−1^. For RRDE measurements, the ring-disk electrode was scanned at a rate of 10 mV·s^−1^ and the ring potential was constant at 0.5 V (0.1 M KOH electrolyte), 1.2 V (0.1 M HClO_4_ electrolyte) vs. RHE. The electron transfer number (n) was determined with the following equations:

(1) $$\begin{array}{c} n=4\times \frac{I_{d}}{I_{d}+I_{r}/N} \end{array}$$

where* I*_*d*_ is the disk current,* I*_*r*_ is the ring current, and* N* = 0.4 is the current collection efficiency of the Pt ring.

## Data Availability

All data needed to evaluate the conclusions in the paper are present in the paper and the Supplementary Materials. Additional data related to this paper may be requested from the authors.

## Supplementary Materials

Supplementary Materials S1: materials and instrumentation. Figure S1: schematic illustration of synthesis of PANI@PCN-224(Fe)-900 and PCN-224(Fe)-900 catalysts. Figure S2: (a) N_2_ adsorption-desorption isotherms and (b) the pore size distribution (PSD) profiles of PCN-224(Fe) and PANI@PCN-224(Fe) based on NL-DFT method. Figure S3: FT-IR spectra of PANI, PCN-224(Fe), and PANI@PCN-224(Fe). Figure S4: UV-Vis spectra of PANI, PANI@PCN-224(Fe), and PCN-224(Fe). Figure S5: PXRD of PCN-224(Fe)-900 and PANI@PCN-224(Fe)-900 before acid etching. Figure S6: Nyquist plots of PANI-900,* X*-PANI@PCN-224(Fe)-900, and PCN-224(Fe)-900 over the frequency range from 100 kHz to 10 mHz. Figure S7: the pore size distribution (PSD) profiles of PCN-224(Fe)-900 and PANI@PCN-224(Fe)-900 based on NL-DFT method. Figure S8: the TEM images of (a) PCN-224(Fe)-900, (b) PANI-900, and (c) 15%-PANI@PCN-224(Fe)-900. Figure S9: high-resolution TEM (HRTEM) of PANI@PCN-224(Fe)-900. Figure S10: the survey XPS spectra of PANI-900, PCN-224(Fe)-900, 15% PANI@PCN-224(Fe)-900, and PANI@PCN-224(Fe)-900. Figure S11: high-resolution N 1s spectra of (a) PANI-900 and (b) PCN-224(Fe)-900. (c) High-resolution Fe 2p spectra of PANI@PCN-224(Fe)-900. (d) Total nitrogen content of different samples. Figure S12: high-resolution N 1s spectra of (a) 15%-PANI@PCN-224(Fe)-900 and (b) PANI@PCN-224(Fe)-900. (c) Nitrogen configurations of 15%-PANI@PCN-224(Fe)-900 and PANI@PCN-224(Fe)-900. Figure S13: methanol-crossover effects test of PANI@PCN-224(Fe)-900 and Pt/C in 0.1 M KOH. Figure S14: electron transfer number of different samples obtained from the RRDE curves 0.1 M HClO_4_. Figure S15: methanol-crossover effects test of PANI@PCN-224(Fe)-900 and Pt/C in 0.1 M HClO_4_. Figure S16: LSVs of PCN-224-900, PCN-224(Fe)-900, and PANI@PCN-224(Fe)-900 in (a) 0.1 M KOH and (b) 0.1 M HClO_4_. Table S1: the N contents of various samples obtained through elemental analysis (EA). Table S2: the Fe contents of various samples obtained through inductively coupled plasma atomic emission spectroscopy (ICP-AES) analysis. Table S3: comparison of ORR catalytic performances in 0.1M KOH between PANI@PCN-224(Fe)-900 and other noble-metal-free electrocatalysts. Table S4: comparison of ORR catalytic performances in 0.1M HClO_4_ between PANI@PCN-224(Fe)-900 and other noble-metal-free electrocatalysts.
