# Supplementary material for: Migration-Prevention Strategy to Fabricate Single-Atom Fe Implanted N-Doped Porous Carbons for Efficient Oxygen Reduction
Source: Research (Wash D C). 2019 Aug 22;2019:1768595. doi: 10.34133/2019/1768595 (PMC6750073; doi:10.34133/2019/1768595)
Supplement: Supplementary Materials — S1: materials and instrumentation. Figure S1: schematic illustration of synthesis of PANI@PCN-224(Fe)-900 and PCN-224(Fe)-900 catalysts. Figure S2: (a) N2 adsorption-desorption isotherms and (b) the pore size distribution (PSD) profiles of PCN-224(Fe) and PANI@PCN-224(Fe) based on NL-DFT method. Figure S3: FT-IR spectra of PANI, PCN-224(Fe), and PANI@PCN-224(Fe). Figure S4: UV-Vis spectra of PANI, PANI@PCN-224(Fe), and PCN-224(Fe). Figure S5: PXRD of PCN-224(Fe)-900 and PANI@PCN-224(Fe)-900 before acid etching. Figure S6: Nyquist plots of PANI-900, X-PANI@PCN-224(Fe)-900, and PCN-224(Fe)-900 over the frequency range from 100 kHz to 10 mHz. Figure S7: the pore size distribution (PSD) profiles of PCN-224(Fe)-900 and PANI@PCN-224(Fe)-900 based on NL-DFT method. Figure S8: the TEM images of (a) PCN-224(Fe)-900, (b) PANI-900, and (c) 15%-PANI@PCN-224(Fe)-900. Figure S9: high-resolution TEM (HRTEM) of PANI@PCN-224(Fe)-900. Figure S10: the survey XPS spectra of PANI-900, PCN-224(Fe)-900, 15% PANI@PCN-224(Fe)-900, and PANI@PCN-224(Fe)-900. Figure S11: high-resolution N 1s spectra of (a) PANI-900 and (b) PCN-224(Fe)-900. (c) High-resolution Fe 2p spectra of PANI@PCN-224(Fe)-900. (d) Total nitrogen content of different samples. Figure S12: high-resolution N 1s spectra of (a) 15%-PANI@PCN-224(Fe)-900 and (b) PANI@PCN-224(Fe)-900. (c) Nitrogen configurations of 15%-PANI@PCN-224(Fe)-900 and PANI@PCN-224(Fe)-900. Figure S13: methanol-crossover effects test of PANI@PCN-224(Fe)-900 and Pt/C in 0.1 M KOH. Figure S14: electron transfer number of different samples obtained from the RRDE curves 0.1 M HClO4. Figure S15: methanol-crossover effects test of PANI@PCN-224(Fe)-900 and Pt/C in 0.1 M HClO4. Figure S16: LSVs of PCN-224-900, PCN-224(Fe)-900, and PANI@PCN-224(Fe)-900 in (a) 0.1 M KOH and (b) 0.1 M HClO4. Table S1: the N contents of various samples obtained through elemental analysis (EA). Table S2: the Fe contents of various samples obtained through inductively coupled plasma atom [file 1768595.f1.zip › 1768595.f1/1768595_SupplDesc.docx]

**Figure S1.** Schematic illustration of synthesis of PANI@PCN-224(Fe)-900 and PCN-224(Fe)-900 catalysts. **Figure S2.** (a) N_2_ adsorption-desorption isotherms and (b) the pore size distribution (PSD) profiles of PCN-224(Fe), PANI@PCN-224(Fe) based on NL-DFT method.

**Figure S3.** FT-IR spectra of PANI, PCN-224(Fe) and PANI@PCN-224(Fe) .

**Figure S4.** UV–vis spectra of PANI, PANI@PCN-224(Fe) and PCN-224(Fe).

**Figure S5.** PXRD of PCN-224(Fe)-900 and PANI@PCN-224(Fe)-900 before acid etching.

**Figure S6.** Nyquist plots of PANI-900, *X*-PANI@PCN-224(Fe)-900, PCN-224(Fe)-900 over the frequency range from 100 kHz to 10 mHz.

**Figure S7.** The pore size distribution (PSD) profiles of PCN-224(Fe)-900, PANI@PCN-224(Fe)-900 based on NL-DFT method. **Figure S8.** The TEM images of (a) PCN-224(Fe)-900, (b)PANI-900 and (c) 15%-PANI@PCN-224(Fe)-900.

**Figure S9.** High-resolution TEM (HRTEM) of PANI@PCN-224(Fe)-900.

**Figure S10.** The survey XPS spectra of PANI-900, PCN-224(Fe)-900, 15% PANI@PCN-224(Fe)-900 and PANI@PCN-224(Fe)-900.

**Figure S11.** High-resolution N 1s spectra of (a) PANI-900, (b) PCN-224(Fe)-900. (c) High-resolution Fe 2p spectra of PANI@PCN-224(Fe)-900. (d) Total nitrogen content of different samples.

**Figure S12.** High-resolution N 1s spectra of (a) 15%-PANI@PCN-224(Fe)-900 and (b) PANI@PCN-224(Fe)-900. (c) Nitrogen configurations of 15%-PANI@PCN-224(Fe)-900 and PANI@PCN-224(Fe)-900.

**Figure S13.** Methanol-crossover effects test of PANI@PCN-224(Fe)-900 and Pt/C in 0.1 M KOH.

**Figure S14.** Electron transfer number of different samples obtained from the RRDE curves 0.1 M HClO_4_.

**Figure S15.** Methanol-crossover effects test of PANI@PCN-224(Fe)-900 and Pt/C in 0.1 M HClO_4_.

**Figure S16.** LSVs of PCN-224-900, PCN-224(Fe)-900 and PANI@PCN-224(Fe)-900 in (a) 0.1 M KOH and (b) 0.1 M HClO_4_.

**Table S1**. The N contents of various samples obtained through elemental analysis (EA).

**Table S2**. The Fe contents of various samples obtained through inductively coupled plasma atomic emission spectroscopy (ICP-AES) analysis.

**Table S3.** Comparison of ORR catalytic performances in 0.1M KOH between PANI@PCN-224(Fe)-900 and other noble-metal-free electrocatalysts.

**Table S4.** Comparison of ORR catalytic performances in 0.1M HClO_4_ between PANI@PCN-224(Fe)-900 and other noble-metal-free electrocatalysts.
