# Supplementary material for: Migration-Prevention Strategy to Fabricate Single-Atom Fe Implanted N-Doped Porous Carbons for Efficient Oxygen Reduction
Source: Research (Wash D C). 2019 Aug 22;2019:1768595. doi: 10.34133/2019/1768595 (PMC6750073; doi:10.34133/2019/1768595)
Supplement: Supplementary Materials — S1: materials and instrumentation. Figure S1: schematic illustration of synthesis of PANI@PCN-224(Fe)-900 and PCN-224(Fe)-900 catalysts. Figure S2: (a) N2 adsorption-desorption isotherms and (b) the pore size distribution (PSD) profiles of PCN-224(Fe) and PANI@PCN-224(Fe) based on NL-DFT method. Figure S3: FT-IR spectra of PANI, PCN-224(Fe), and PANI@PCN-224(Fe). Figure S4: UV-Vis spectra of PANI, PANI@PCN-224(Fe), and PCN-224(Fe). Figure S5: PXRD of PCN-224(Fe)-900 and PANI@PCN-224(Fe)-900 before acid etching. Figure S6: Nyquist plots of PANI-900, X-PANI@PCN-224(Fe)-900, and PCN-224(Fe)-900 over the frequency range from 100 kHz to 10 mHz. Figure S7: the pore size distribution (PSD) profiles of PCN-224(Fe)-900 and PANI@PCN-224(Fe)-900 based on NL-DFT method. Figure S8: the TEM images of (a) PCN-224(Fe)-900, (b) PANI-900, and (c) 15%-PANI@PCN-224(Fe)-900. Figure S9: high-resolution TEM (HRTEM) of PANI@PCN-224(Fe)-900. Figure S10: the survey XPS spectra of PANI-900, PCN-224(Fe)-900, 15% PANI@PCN-224(Fe)-900, and PANI@PCN-224(Fe)-900. Figure S11: high-resolution N 1s spectra of (a) PANI-900 and (b) PCN-224(Fe)-900. (c) High-resolution Fe 2p spectra of PANI@PCN-224(Fe)-900. (d) Total nitrogen content of different samples. Figure S12: high-resolution N 1s spectra of (a) 15%-PANI@PCN-224(Fe)-900 and (b) PANI@PCN-224(Fe)-900. (c) Nitrogen configurations of 15%-PANI@PCN-224(Fe)-900 and PANI@PCN-224(Fe)-900. Figure S13: methanol-crossover effects test of PANI@PCN-224(Fe)-900 and Pt/C in 0.1 M KOH. Figure S14: electron transfer number of different samples obtained from the RRDE curves 0.1 M HClO4. Figure S15: methanol-crossover effects test of PANI@PCN-224(Fe)-900 and Pt/C in 0.1 M HClO4. Figure S16: LSVs of PCN-224-900, PCN-224(Fe)-900, and PANI@PCN-224(Fe)-900 in (a) 0.1 M KOH and (b) 0.1 M HClO4. Table S1: the N contents of various samples obtained through elemental analysis (EA). Table S2: the Fe contents of various samples obtained through inductively coupled plasma atom [file 1768595.f1.zip › 1768595.f1/ESI-Revised.docx]

Supporting Information

**Migration-Prevention Strategy to Fabricate Single-Atom Fe Implanted N-doped Porous Carbons for Efficient Oxygen Reduction**

*Dong-Li Meng*^1^, *Jing Shi*^2^*, Jun Luo*^2^*, Chun-Hui Chen*^1^, *Jun-Dong Yi*^1^, *Qiao Wu*^1^, *Jun Liang*^1^, *Yuan-Biao Huang*^1,3^***, *and Rong Cao*^1,3^***

*^1^* State Key Laboratory of Structural Chemistry, Fujian Institute of Research on the Structure of Matter, Chinese Academy of Sciences, Fuzhou 350002, China

*^2^* Center for Electron Microscopy, Institute for New Energy Materials and Low-Carbon Technologies, School of Materials, Tianjin, University of Technology, Tianjin 300384, China

*^3^* University of the Chinese Academy of Sciences, Beijing 100049, China

^*^Correspondence should be addressed to Yuan-Biao Huang; [ybhuang@fjirsm.ac.cn](mailto:ybhuang@fjirsm.ac.cn) and Rong Cao; [rcao@fjirsm.ac.cn](mailto:rcao@fjirsm.ac.cn)

**S1: Materials and Instrumentation:**

All reagents and chemicals were purchased from commercial sources and used as obtained without further purification. methyl 4-formylbenzoate (98%, J﹠K), propionic acid (99%, J﹠K), pyrrole (99%, TCI), zirconyl chloride octahydrate (ZrOCl_2_·8H_2_O) (98%, Adamas), FeCl_2_·4H_2_O (99%, Sigma-Aldrich), commercial Pt/C (20 wt% metal, Alfa Aesar), nafion solution (5% in lower aliphatic alcohols and water, Sigma-Aldrich) were purchased from the commercial corporations. FT-IR spectra were recorded on a Vertex70 spectrometer with a resolution of 0.2 cm^-1^ using KBr pellets in the range of 400–4000 cm^−1^. UV-visible spectra were recorded on a Lambda 950 UV-Vis-NIR spectrometer. The samples were uniformly smeared onto the plate with barium sulfate base. Powder X-ray diffraction (PXRD) patterns were recorded on a Miniflex 600 diffractometer using Cu Κα radiation (λ = 0.154 nm) with a scan speed of 2° min^−1^ at room temperature. Transmission electron microscope (TEM) images were recorded by a FEIT 20 working at 200 kV. Aberration-corrected High-angle annular dark-field scanning transmission electron microscopy (HAADF-STEM) images and the EDS of samples were performed with a Titan Cubed Themis G2 300 (FEI) high-resolution transmission electron microscope operated at 200 kV. The Raman spectrum was tested on a Labram HR800 Evolution over a range of 300-2000 cm^-1^. N_2_ adsorption-desorption isotherm were measured using Micromeritics ASAP 2460 instrument at 77 K. Prior to nitrogen adsorption/desorption measurement, the samples were evacuated and activated at 120 °C for 10 h by molecular pump. The Nyquist plots were obtained by the electrochemical impedance spectroscopy (EIS) measurement. It was performed by applying AC voltage with 5 mV amplitude in a frequency range from 0.01 Hz to 100 kHz under open circuit potential condition. X-ray photoelectron spectroscopy (XPS) measurements were performed on an ESCALAB 250Xi X-ray photoelectron spectrometer (Thermo Fisher) using an Al Ka source (15 kV, 10 mA). XAFS spectraat the Fe K-edge (7112 eV) were conducted at the beamline BL14W1 station of the Shanghai Synchrotron Radiation Facility, China. The Fe K-edge XANES data were recorded in a transmission mode and Fe foil was used as references. Elemental analyses of N were carried out on an Elementar Vario EL III analyzer. The Fe content was characterized by inductively coupled plasma atomic emission spectroscopy (ICP-AES) on an Ultima 2 analyzer (Jobin Yvon).

^
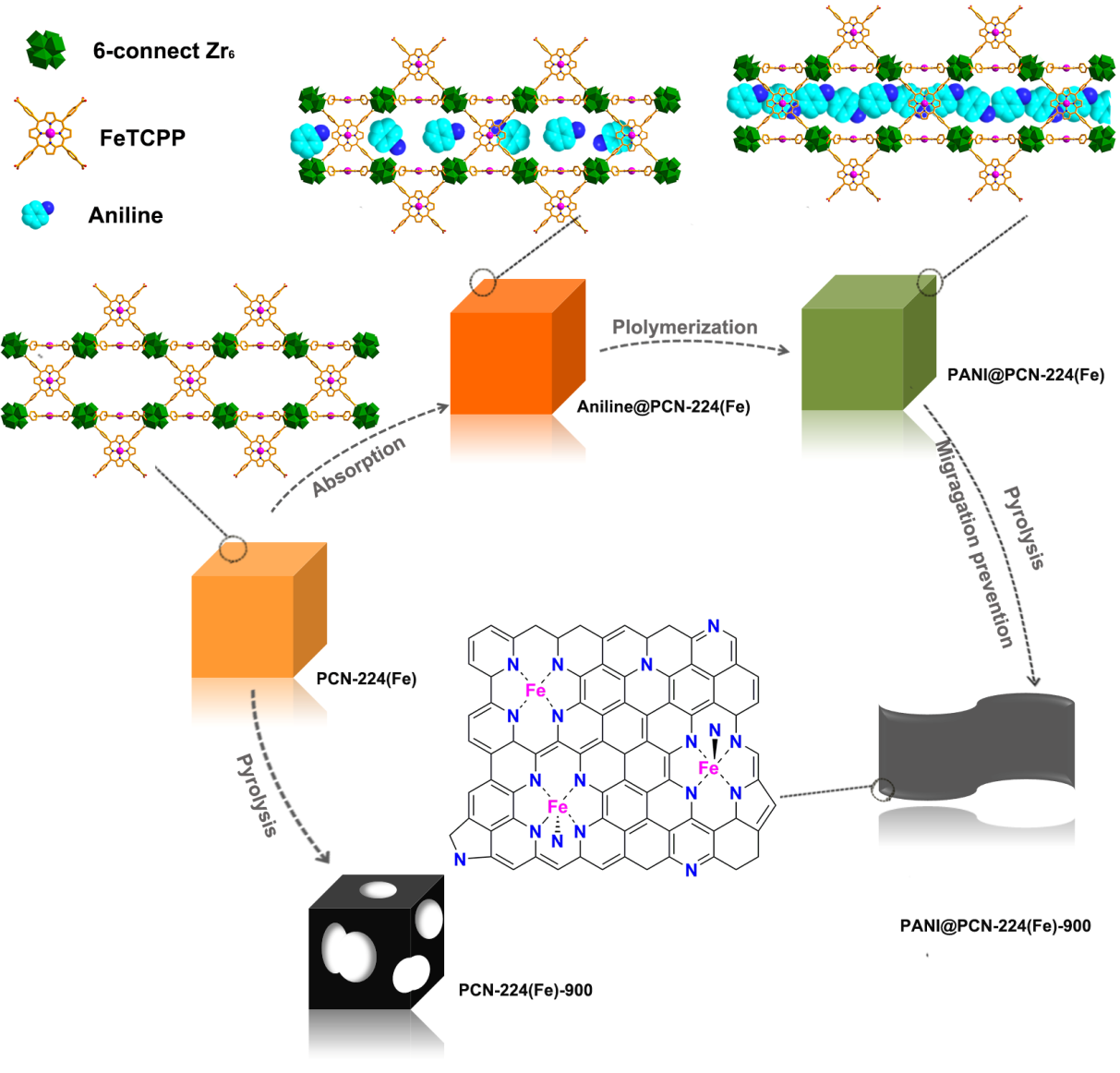
^

**Figure S1.** Schematic illustration of synthesis of PANI@PCN-224(Fe)-900 and PCN-224(Fe)-900 catalysts.


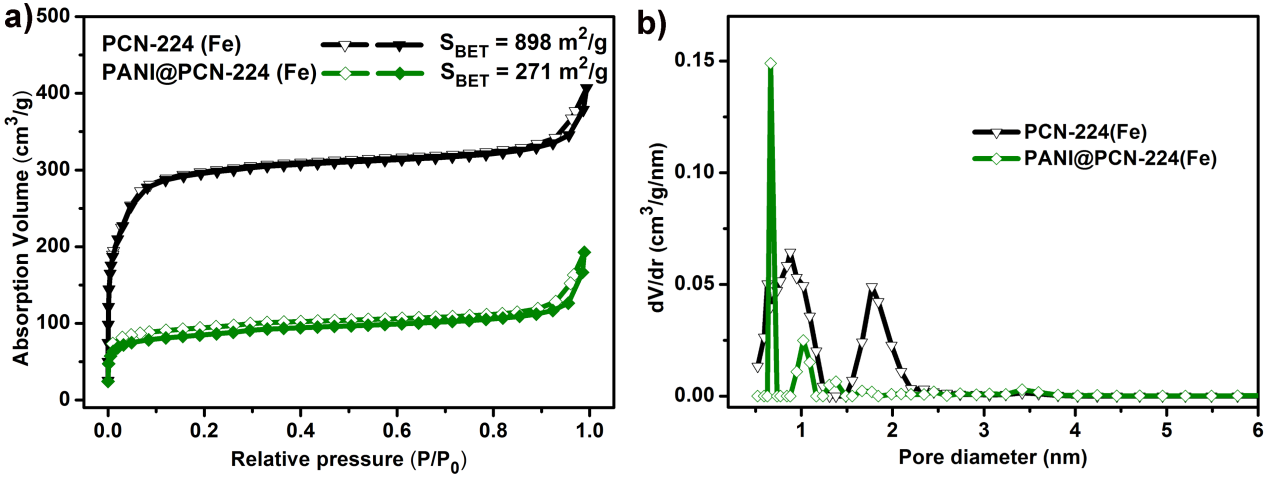


**Figure S2.** (a) N_2_ adsorption-desorption isotherms and (b) the pore size distribution (PSD) profiles of PCN-224(Fe), PANI@PCN-224(Fe) based on NL-DFT method.


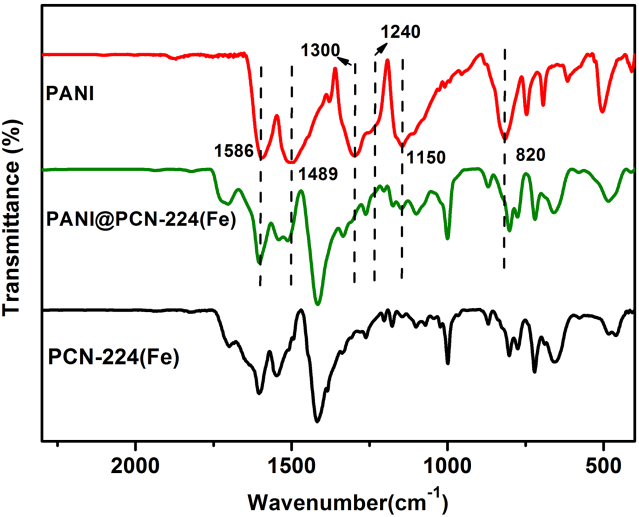


**Figure S3.** FT-IR spectra of PANI, PCN-224(Fe) and PANI@PCN-224(Fe) .


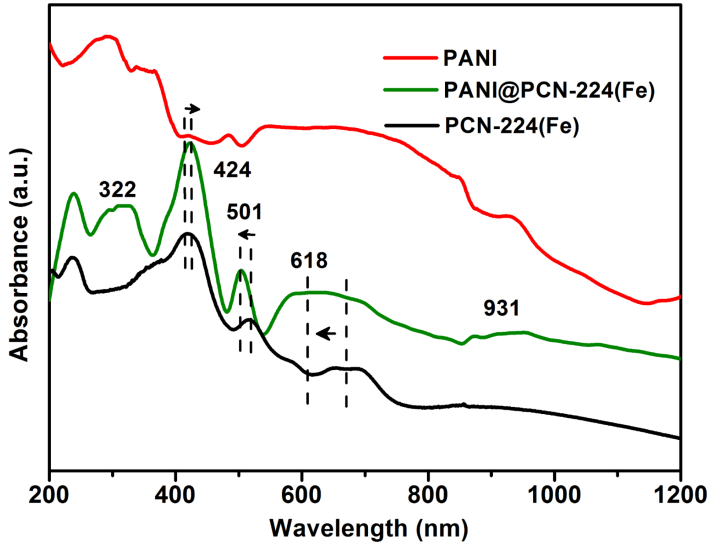


**Figure S4.** UV–vis spectra of PANI, PANI@PCN-224(Fe) and PCN-224(Fe).

**
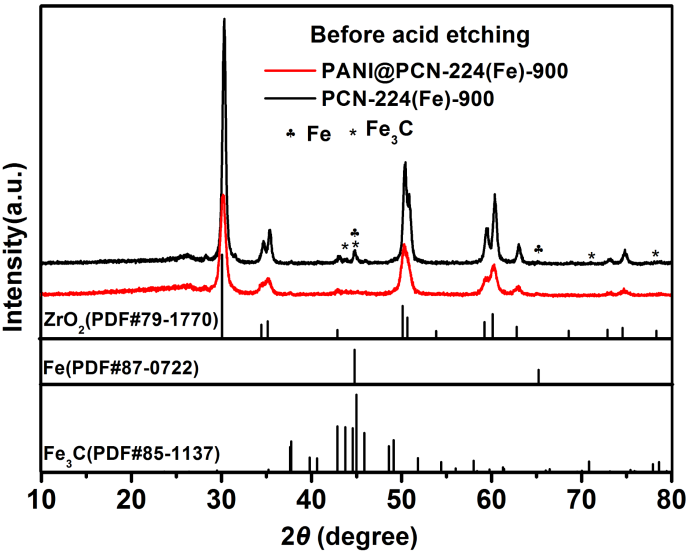
**

**Figure S5.** PXRD of PCN-224(Fe)-900 and PANI@PCN-224(Fe)-900 before acid etching.


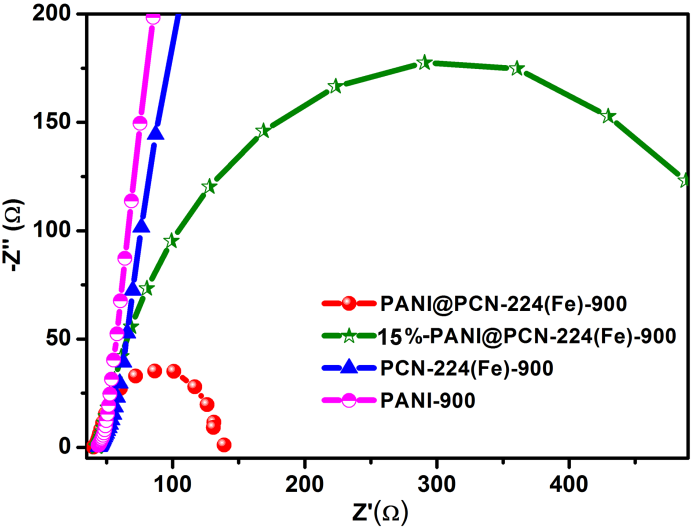


**Figure S6.** Nyquist plots of PANI-900, *X*-PANI@PCN-224(Fe)-900, PCN-224(Fe)-900 over the frequency range from 100 kHz to 10 mHz.


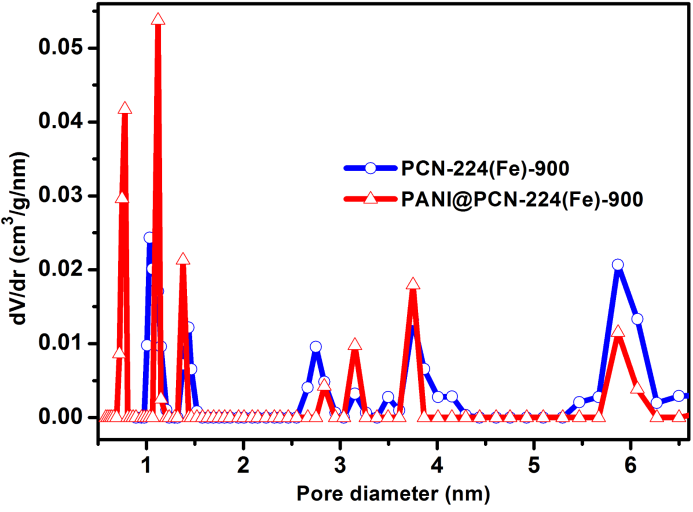


**Figure S7.** The pore size distribution (PSD) profiles of PCN-224(Fe)-900, PANI@PCN-224(Fe)-900 based on NL-DFT method.

^
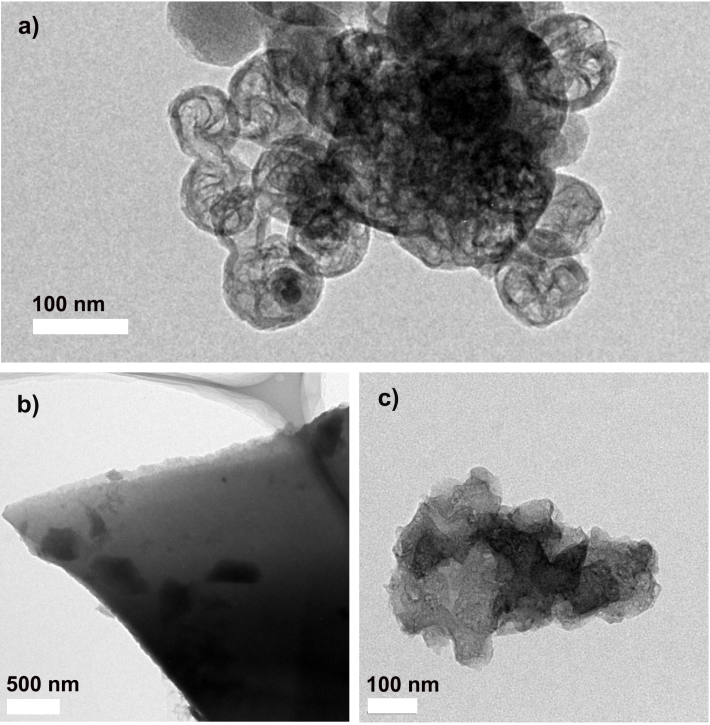
^

**Figure S8.** The TEM images of (a) PCN-224(Fe)-900, (b)PANI-900 and (c) 15%-PANI@PCN-224(Fe)-900.


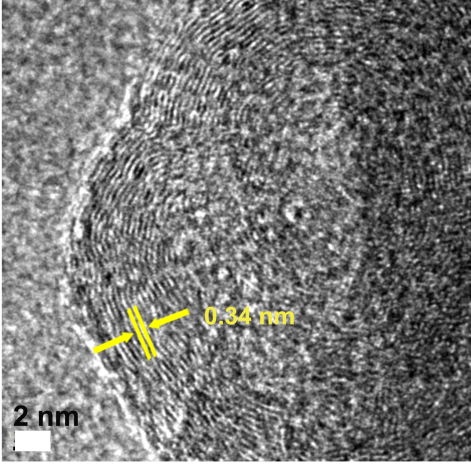


**Figure S9.** High-resolution TEM (HRTEM) of PANI@PCN-224(Fe)-900.


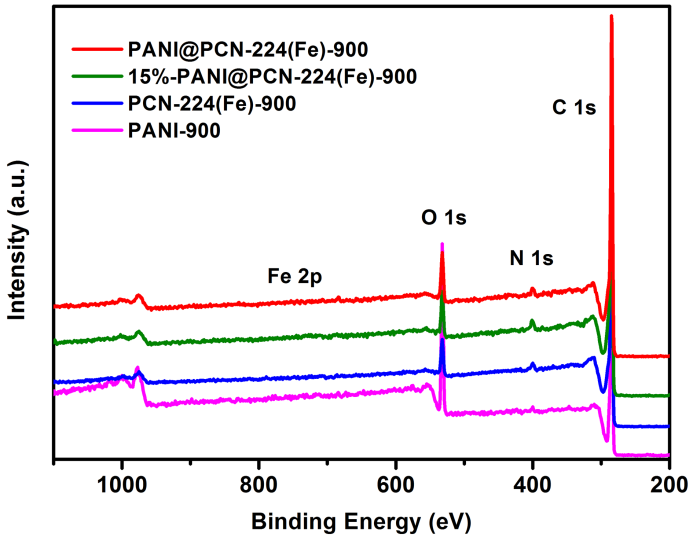


**Figure S10.** The survey XPS spectra of PANI-900, PCN-224(Fe)-900, 15% PANI@PCN-224(Fe)-900 and PANI@PCN-224(Fe)-900.


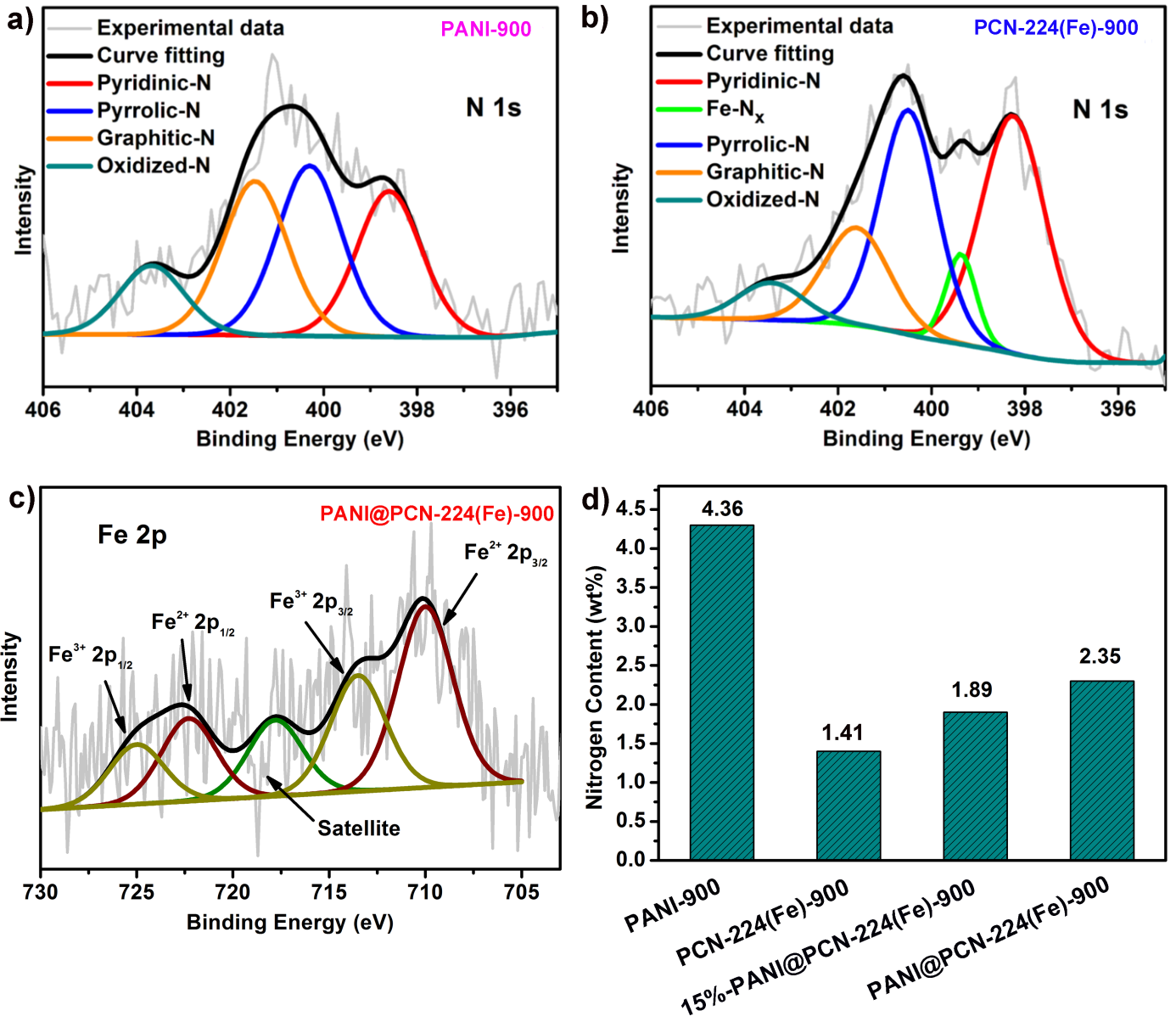


**Figure S11.** High-resolution N 1s spectra of (a) PANI-900, (b) PCN-224(Fe)-900. (c) High-resolution Fe 2p spectra of PANI@PCN-224(Fe)-900. (d) Total nitrogen content of different samples.


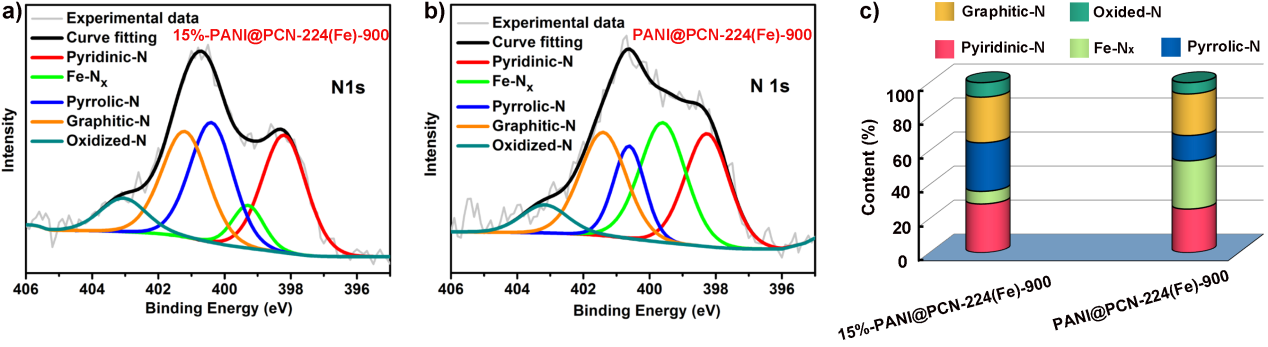


**Figure S12.** High-resolution N 1s spectra of (a) 15%-PANI@PCN-224(Fe)-900 and (b) PANI@PCN-224(Fe)-900. (c) Nitrogen configurations of 15%-PANI@PCN-224(Fe)-900 and PANI@PCN-224(Fe)-900.


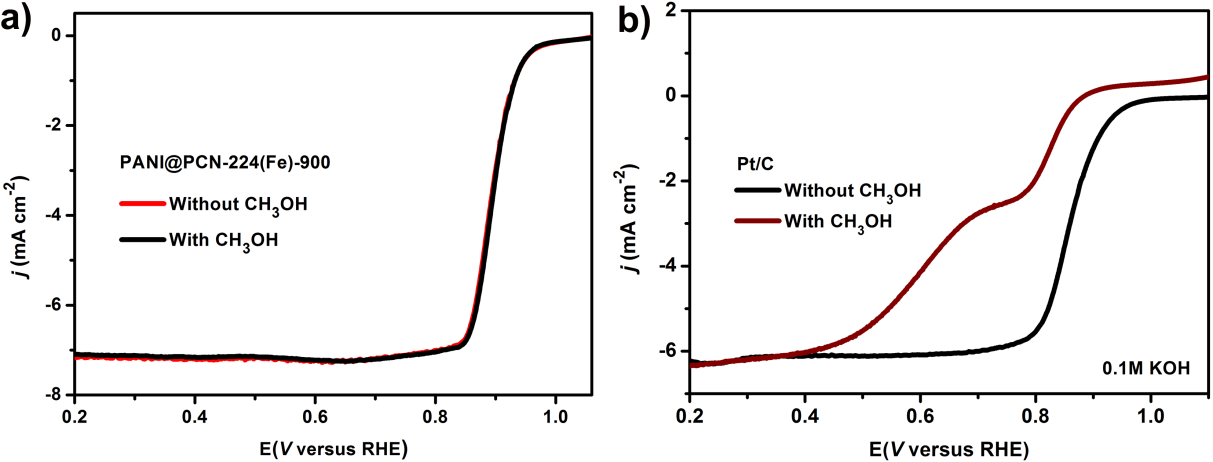


**Figure S13.** Methanol-crossover effects test of PANI@PCN-224(Fe)-900 and Pt/C in 0.1 M KOH.


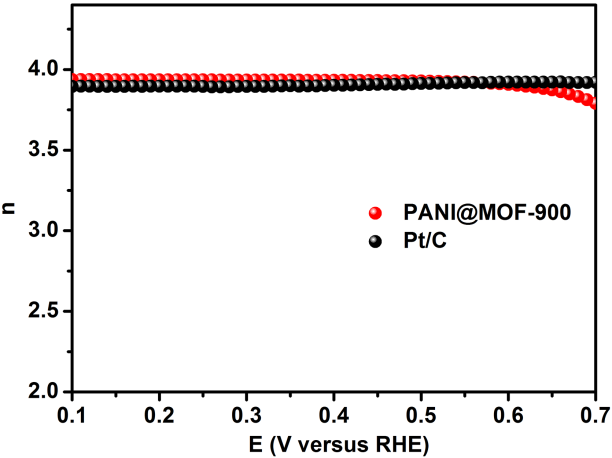


**Figure S14.** Electron transfer number of different samples obtained from the RRDE curves 0.1 M HClO_4_.


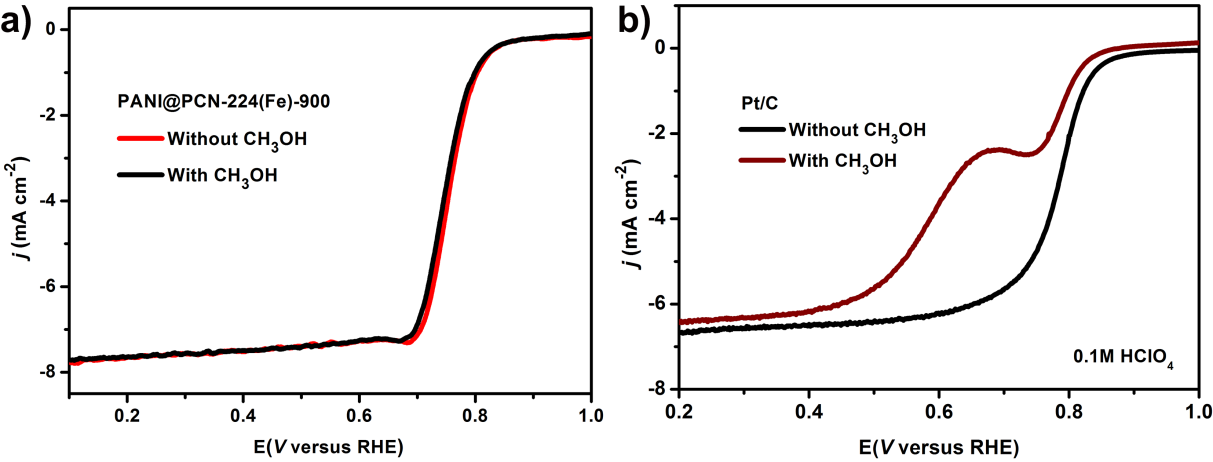


**Figure S15.** Methanol-crossover effects test of PANI@PCN-224(Fe)-900 and Pt/C in 0.1 M HClO_4_.


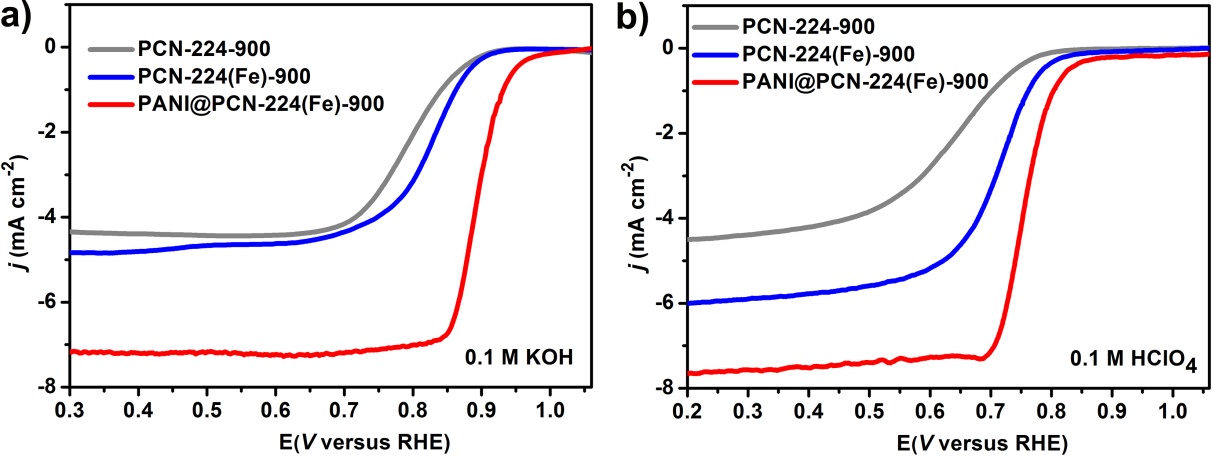


**Figure S16.** LSVs of PCN-224-900, PCN-224(Fe)-900 and PANI@PCN-224(Fe)-900 in (a) 0.1 M KOH and (b) 0.1 M HClO_4_.

**Table S1**. The N contents of various samples obtained through elemental analysis (EA).

| **Sample** | **PANI-900** | **PCN-224(Fe)-900** | **15%-PANI@PCN-224(Fe)-900** | **PANI@PCN-224(Fe)-900** |
| --- | --- | --- | --- | --- |
| **N content**  **(wt%)** | **7.73** | **1.39** | **1.83** | **2.57** |

**Table S2**. The Fe contents of various samples obtained through inductively coupled plasma atomic emission spectroscopy (ICP-AES) analysis.

| **Sample** | **PCN-224(Fe)-900** | **15%-PANI@PCN-224(Fe)-900** | **PANI@PCN-224(Fe)-900** |
| --- | --- | --- | --- |
| **Fe content (wt%)** | **0.27** | **0.35** | **1.89** |

**Table S3.** Comparison of ORR catalytic performances in 0.1M KOH between PANI@PCN-224(Fe)-900 and other noble-metal-free electrocatalysts.

|  | Class |  |  | Catalyst | Half-wave potential  (V *vs.* RHE) | Onset potential  (V *vs.* RHE) | Ref. |
| --- | --- | --- | --- | --- | --- | --- | --- |
| Fe-based catalysts  Fe-based catalysts | | | | PANI@PCN-224(Fe)-900 | 0.893 | 1.01 | This work |
|  |  |  |  | Pt/C | 0.856 | 0.973 | This work |
|  |  |  |  | Fe@Aza-PON | 0.839 | <0.95 | *J. Am. Chem. Soc.* 2018*, 140,* 1737–1742 |
|  |  |  |  | FeBNC-800 | 0.838 | 0.968 | *ACS Energy Lett.* 2018, *3*, 252-260 |
|  |  |  |  | FeSAs/PTF-600 | 0.87 | 1.01 | *ACS Energy Lett*. 2018, *3*, 883-889 |
|  |  |  |  | FeSA-N-C | 0.89 | 1.00 | *Angew. Chem. Int. Ed.* 2018, *57*, 8525 -8529 |
|  |  |  |  | Fe_0.3_Co_0.7_/NC | 0.88 | ~0.97 | *Adv. Funct. Mater.* 2018, *28*, 1706738 |
|  |  |  |  | Fe2-Z8-C | 0.871 | 0.985 | *Angew. Chem. Int. Ed.* 2018, *57*, 1204-1208 |
|  |  |  |  | PPy/FeTCPP/Co | 0.86 | 1.01 | *Adv. Funct. Mater.* 2017, *27*, 1606497 |
|  |  |  |  | NPME with 33.3 wt% FeTMPPCl | 0.87 | 0.936 | *Adv. Funct. Mater.* 2017*, 27*,1604356 |
|  |  |  |  | NH_3_-Fe_0.25_-N/C-900 | 0.865 | 1.02 | *Nano Energy* 2017*, 36,* 286-297 |
|  |  |  |  | Fe-N-SCCFs | 0.883 | 1.03 | *Nano Lett.* 2017, *17,* 2003-2009 |
|  |  |  |  | CNT/PC | 0.88 | 0.95 | *J. Am. Chem. Soc.* 2016, *138*, 15046-15056 |
|  |  |  |  | Fe-N/C-800 | ~0.80 | 0.98 | *J. Am. Chem. Soc.,* 2015, *137*, 5555–5562 |
|  |  |  |  | FePhen@MOF-ArNH_3_ | 0.86 | 1.03 | *Nat. Commun.* 2015, 6, 7343 |
|  |  |  |  | Fe-N-CNFs | 0.81 | 0.93 | *Angew .Chem. Int. Ed.* 2015, *54*, 8179-8183 |
|  |  |  |  | Fe−N/C-800 | 0.81 | 0.92 | *J. Am. Chem. Soc.* 2014, *136*, 11027-11033 |
|  |  |  |  | Fe_3_C/C-800 | 0.83 | 1.05 | *Angew. Chem.* 2014*, 126,* 3749 –3753 |
|  |  |  |  | Fe-PANI/C-Mela | 1.01 | 0.88 | *ACS Catal.* 2014, 4, 3797−3805 |
|  |  |  |  | FeIM/ZIF-8 | 0.75 | 0.92 | *Chem. Sci.* 2012, *3*, 3200-3205 |
|  |  |  |  | PANI-Fe-C | 0.81 | 0.91 | *Science* 2011*, 332,* 443-447 |
| Other non-precious  metal catalysts | | | | N-CNTs-650 | 0.85 | 0.94 | *J. Am. Chem. Soc.* 2017,*139*, 8212-8221 |
|  |  |  |  | Co SAs/N-C(900) | 0.881 | 0.982 | *Angew. Chem. Int. Ed.* 2016*, 55,* 10800-10805 |
|  |  |  |  | Co-NC | 0.83 | ~0.9 | *Angew. Chem. Int. Ed.* 2016, *55*, 4087-4091 |
|  |  |  |  | Co,N-CNF | 0.81 | 0.88 | *Adv. Mater.* 2016, *28*, 1668-1674 |
|  |  |  |  | Carbon nanotube  frameworks | 0.87 | 0.97 | *Nat. Energy* 2016, *1*, 15006 |
|  |  |  |  | N, P-CGHNs | 0.81 | 0.93 | *Adv. Mater.* 2016, 28,4606-4613 |
|  |  |  |  | CoO/C | 0.77 | 0.85 | *ACS Catal.* 2014, *4*,2998-3001 |

**Table S4.** Comparison of ORR catalytic performances in 0.1M HClO_4_ between PANI@PCN-224(Fe)-900 and other noble-metal-free electrocatalysts.

|  | **Class** |  |  | **Catalyst** | **Half-wave potential**  **(V *vs.* RHE)** | **Onset potential**  **(V *vs.* RHE)** | **Ref.** |
| --- | --- | --- | --- | --- | --- | --- | --- |
| **Fe-based catalysts** | | | | **PANI@PCN-224(Fe)-900** | **0.756** | **0.891** | **This work** |
|  |  |  |  | **Pt/C** | **0.783** | **0.907** | **This work** |
|  |  |  |  | Fe@Aza-PON | 0.541 | <0.75 | *J. Am. Chem. Soc.,* **2018***, 140,* 1737–1742 |
|  |  |  |  | NC-CNT-1000 | <0.40 | <0.75 | Adv. Funct. Mater. **2018**, *28*, 1707284 |
|  |  |  |  | NPME with 50.0 wt% FeTMPPC | 0.748 | 0.824 | *Adv. Funct. Mater.* **2017***, 27*,1604356 |
|  |  |  |  | NH_3_-Fe_0.25_-N/C-900 | 0.710 | 0.935 | *Nano Energy* **2017***, 36,* 286-297 |
|  |  |  |  | CPM-99Fe/C | ~0.75 | <0.88 | *J. Am. Chem. Soc.* **2015**, *137*, 2235-2238 |
|  |  |  |  | CPANI-Fe-NaCl | <0.73 | <0.95 | *J. Am. Chem. Soc.* **2015***, 137,* 5414-5420 |
|  |  |  |  | FePhen@MOF-ArNH_3_ | 0.77 | 0.93 | *Nat. Commun.* **2015**, *6*, 7343 |
|  |  |  |  | Fe-N-doped Hollow Carbon Nanoshells | ~0.74 | ~0.85 | *ACS Catal.* **2015**, *5*,3887-3893 |
|  |  |  |  | Fe-N/C-800 | ~0.60 | 0.82 | *J. Am. Chem. Soc.* **2014***, 136*, 11027-11033 |
|  |  |  |  | Fe_3_C/C-700 | 0.73 | <0.83 | *Angew. Chem. Int. Ed.* **2014**, *53*, 3675-3679 |
|  |  |  |  | Fe_3_C/C-700 | 0.73 | 0.90 | *Angew. Chem.* **2014***, 126,* 3749 –3753 |
|  |  |  |  | Fe-PANI/C-Mela | 0.76 | 0.91 | *ACS Catal.* **2014**, 4, 3797−3805 |
|  |  |  |  | Fe-N/C-800 | ~0.60 | 0.77 | *J. Am. Chem. Soc.,* **2015**, *137*, 5555–5562 |
| Other non-precious  metal catalysts | | | | bNGr | <0.70 | <0.88 | *J. Am. Chem. Soc.* **2014**, *136*, 9070-9077 |
|  |  |  |  | N-G-CNT | <0.5 | <0.8 | *Sci. Adv.* **2015**, *1*, e1400129 |
|  |  |  |  | N, P-CGHNs | 0.68 | 0.90 | *Adv. Mater.* **2016**, *28*, 4606-4613 |
|  |  |  |  | N,P-mesoporous nanocarbon | 0.62 | 0.83 | *Nature Nanotech.* **2015**, *10*,  444 |
|  |  |  |  | Co-N-C | 0.76 | ~0.9 | *ACS Catal.* **2015**, *5*,7068-7076 |
